# Progressive optic atrophy in a retinal ganglion cell-specific mouse model of complex I deficiency

**DOI:** 10.1038/s41598-020-73353-0

**Published:** 2020-10-01

**Authors:** Luyu Wang, Mikael Klingeborn, Amanda M. Travis, Ying Hao, Vadim Y. Arshavsky, Sidney M. Gospe

**Affiliations:** 1 Department of Ophthalmology, Box 3712 Med Center, Duke University, 2351 Erwin Road, Durham, NC 27710 USA; 2 Department of Ophthalmology and Visual Sciences, University of Michigan, Ann Arbor, MI 48105 USA; 3 Department of Pharmacology and Cancer Biology, Duke University School of Medicine, Durham, NC 27710 USA

**Keywords:** Optic nerve diseases, Hereditary eye disease, Neurodegeneration

## Abstract

Optic atrophy resulting from retinal ganglion cell (RGC) degeneration is a prominent ocular manifestation of mitochondrial dysfunction. Although transgenic mice lacking the mitochondrial complex I accessory subunit NDUFS4 develop early-onset optic atrophy, severe systemic mitochondrial dysfunction leads to very early death and makes this mouse line impractical for studying the pathobiology of mitochondrial optic neuropathies. Theoretically, RGC-specific inactivation of *ndufs4* would allow characterization of RGC degeneration over a longer time course, provided that RGC death from mitochondrial dysfunction is a cell-autonomous process. We demonstrate that the vesicular glutamate transporter VGLUT2 may be exploited to drive robust Cre recombinase expression in RGCs without any expression observed in directly neighboring retinal cell types. Deletion of *ndufs4* in RGCs resulted in reduced expression of NDUFS4 protein within the optic nerves of *Vglut2-Cre;ndufs4*^*loxP/loxP*^ mice. RGC degeneration in *Vglut2-Cre;ndufs4*^*loxP/loxP*^ retinas commenced around postnatal day 45 (P45) and progressed to loss of two-thirds of RGCs by P90, confirming that intrinsic complex I dysfunction is sufficient to induce RGC death. The rapidly-developing optic atrophy makes the *Vglut2-Cre;ndufs4*^*loxP/loxP*^ mouse line a promising preclinical model for testing therapies for currently untreatable mitochondrial optic neuropathies such as Leber Hereditary Optic Neuropathy.

## Introduction

Mitochondrial dysfunction contributes to vision loss in many retinal and optic nerve disorders^1–3^, with a small but important subset comprised by heritable mitochondrial diseases^4^. Chief among these is Leber Hereditary Optic Neuropathy (LHON), a maternally-inherited disease in which rapid degeneration of retinal ganglion cells (RGCs) results in profound vision loss, typically in previously healthy adolescents and young adults^5^. With a prevalence of 1:30,000 to 1:50,000, LHON is likely the most common human disease with a mitochondrial pattern of inheritance^6–8^. It is caused by hypomorphic missense mutations in mitochondrial DNA (mtDNA) encoding subunits of respiratory complex I (NADH:ubiquinone oxidoreductase). This large protein complex found in the mitochondrial inner membrane is composed of seven mtDNA-encoded subunits and approximately 37 nuclear-encoded subunits^9^. Because the loss of function resulting from the mtDNA mutations associated with LHON is usually relatively mild, homoplasmy of the mutations is frequently required for optic neuropathy to develop^10–12^, and the remaining tissues of the body are usually (although not invariably) spared from pathology^13,14^. This is in stark contrast to the severe systemic mitochondrial disease Leigh syndrome, some forms of which also develop from mutations affecting complex I subunits^15^. In the latter cases, the mutations result in more profound dysfunction of mitochondrial respiration than in LHON^16^, leading to generalized weakness, hypotonia and progressive cardiac and respiratory failure, often culminating in death in early childhood. Notably, patients with Leigh syndrome also frequently manifest optic atrophy^17^.

There are currently no satisfactory methods of preventing or treating LHON or other mitochondrial optic neuropathies. Efforts to develop effective pharmacotherapies have been hampered, in part, by the lack of an ideal preclinical animal model for efficient drug testing. Two experimental mouse lines have been constructed which carry heritable mtDNA mutations affecting complex I and have been reported to exhibit LHON-like phenotypes. One mouse line with a homoplasmic point mutation in the complex I subunit ND6 demonstrates loss of ~ 25% of RGC axons within the optic nerve by 24 months of age^18^. The second model, a mouse line carrying a human mtDNA mutation of the ND4 subunit as a mitochondrial episome, demonstrates the onset of retinal electrophysiological abnormalities by 4 months and some thinning of inner retinal layers corresponding to RGCs by 7 months^19^.

Theoretically, a more rapidly progressive optic neuropathy would result from mutations that produce more severe complex I dysfunction than observed in these existing models. For instance, deletion of the nuclear gene *ndufs4*, which encodes an accessory complex I subunit mutated in some forms of Leigh syndrome, results in severe instability of complex I and decreases its enzymatic activity by > 50% in the retina and other tissues^20–22^. Indeed, the germline *ndufs4* knockout mouse (henceforth denoted *ndufs4*^*−/−*^) has been reported to exhibit an early onset of RGC degeneration by postnatal day 42 (P42)^23^. However, the utility of this mouse line as a model of mitochondrial optic neuropathy is greatly limited by its shortened lifespan: the mice proceed to die rapidly after P50 due to progressive encephalomyopathy^22^. Conditional deletion of *ndufs4* within RGCs is a potential strategy to achieve efficient RGC degeneration with a less severe systemic phenotype, although this would require that complex I deficiency intrinsic to RGCs is sufficient to induce degeneration. The high energy demands of RGCs have long been believed to explain their particular susceptibility to mitochondrial dysfunction^24^, yet it remains possible that pathology of other neighboring retinal neurons may play a critical role in inducing RGC degeneration^20,23^. We used a transgenic mouse line expressing Cre recombinase within a subset of glutamatergic neurons to test the hypothesis that RGC degeneration in complex I deficiency is a cell-autonomous process and to characterize the progression of optic atrophy over multiple time points.

## Results

### Histological assessment of *Vglut2*-driven Cre recombinase expression in the retina

**Figure 1:**
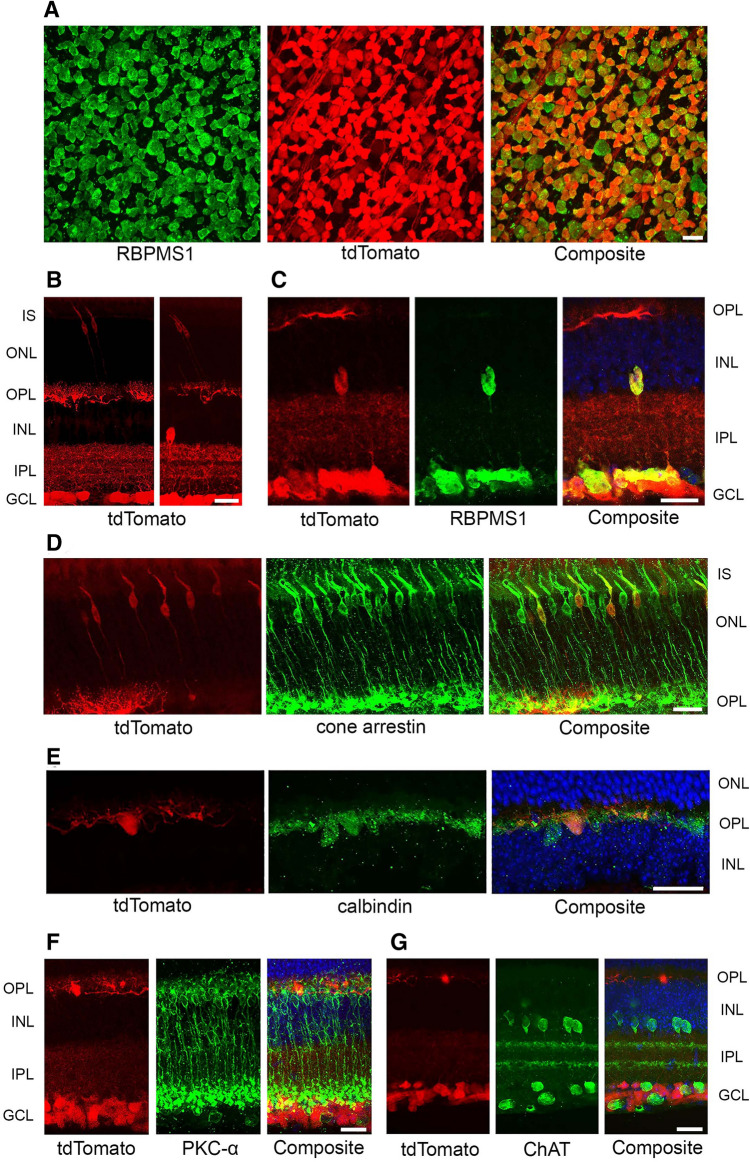
*Vglut2*-driven Cre expression is present in the vast majority of RGCs and a minority of non-RGC retinal neurons. (**A**) Immunolabeling of RGCs with RNA-Binding Protein 1 (RBPMS1) in *Vglut2-Cre;Ai9* retinal flat mounts. Approximately 96% of RBPMS1-positive cells (green) show co-expression of tdTomato (red). (**B**) Two representative images of tdTomato reporter expression in *Vglut2-Cre;Ai9* retinal cross-sections, demonstrating tdTomato expression in cells in the GCL (and their dendritic projections in the IPL), cells in the OPL, and rare photoreceptors with nuclei at the top of the ONL. The right panel shows an example of a solitary tdTomato-positive cell at the base of the INL. (**C**) Co-localization of tdTomato and RBPMS1 identifies the Cre-expressing cell in the INL as a displaced RGC. (**D**,**E**) Co-localization of tdTomato with cone arrestin (**D**) and calbindin (**E**) confirms the identities of tdTomato-positive cells in the ONL and OPL as cones and horizontal cells, respectively. (**F**,**G**) tdTomato is not expressed in rod bipolar cells labeled with PKC-α (**F**) or in starburst amacrine cells labeled with ChAT (**G**). *IS* inner segment, *ONL* outer nuclear layer, *OPL* outer plexiform layer, *INL* inner nuclear layer, *IPL* inner plexiform layer, *GCL* ganglion cell layer. All scale bars, 20 μm.

The vesicular glutamate transporter VGLUT2 is expressed in RGCs^25^ and was previously shown to drive expression of Cre recombinase in virtually all RGCs in a transgenic mouse line with an *IRES-Cre* cassette inserted directly downstream from the *Vglut2* stop codon (henceforth referred to as *Vglut2-Cre*)^26^. However, Cre expression in other retinal neurons in this mouse line has not been reported. By crossing the *Vglut2-Cre* transgenic mouse with the *Ai9* mouse line expressing the fluorescent reporter tdTomato from the Rosa26 locus in a Cre-dependent manner, we evaluated *Vglut2*-driven Cre expression throughout the retina. In retinal flatmounts, 96% ± 0.6% (mean ± SEM) of cells in the ganglion cell layer that labeled with the RGC marker RBPMS1^27^ co-expressed tdTomato (Fig. 1A), and fewer than 1% of tdTomato-expressing cells failed to label for RBPMS1, indicating excellent specificity for RGCs within this retinal layer. Consistent with prior reports describing the expression of endogenous VGLUT2 in the retina^25,28^, only a small minority of retinal neurons outside the ganglion cell layer demonstrated expression of *Vglut2*-driven Cre (Fig. 1B). Retinal cross-sections exhibited tdTomato expression within rare cells in the inner nuclear layer at its junction with the inner plexiform layer; co-labeling with RBPMS1 confirmed that these represent displaced RGCs (Fig. 1C)^27^. Only a small fraction of photoreceptors exhibited tdTomato fluorescence, and these were found to represent a subset of cones based on co-labeling with cone arrestin [10.3% ± 1.2% of cones (mean ± SEM)] (Fig. 1D). A smaller fraction of horizontal cells also expressed tdTomato [6.4% ± 3.7% (mean ± SEM)] (Fig. 1E), consistent with endogenous VGLUT2 expression^25^.

Prior reports have suggested that, in the germline *ndufs4*^*−/−*^ mouse, rod bipolar cells^20^ and starburst amacrine cells^23^ degenerate early and may contribute to the death of RGCs in this model. We co-labeled retinal cross-sections from *Vglut2-Cre;Ai9* mice with PKC-α and ChAT and found no evidence of Cre expression in either rod bipolar cells or starburst amacrine cells, respectively (Fig. 1F,G). Thus, the *Vglut2-Cre* transgenic mouse line provides an opportunity to study the effect of genetic modification of RGCs without any confounding effects from altering directly neighboring cell types.

### RGC somas and axons undergo progressive degeneration in *Vglut2-Cre;ndufs4*^*loxP/loxP*^ mice

Having demonstrated the high degree of specificity for *Vglut2*-driven Cre expression in RGCs histologically, we proceeded to establish a *Vglut2-Cre;ndufs4*^*loxP/loxP*^ transgenic mouse line in order to achieve RGC-specific deletion of *ndufs4*. The systemic phenotype of *ndufs4* deletion in *Vglut2*-expressing glutamatergic neurons was recently described by another group which independently generated *Vglut2-Cre;ndufs4*^*loxP/loxP*^ transgenic mice but did not characterize the retinal phenotype^29^. Consistent with their report, we observed that *Vglut2-Cre;ndufs4*^*loxP/loxP*^ mice are initially healthy but develop progressive weight loss, ataxia, and hindlimb weakness beginning around P60. As only a subset of neurons experience loss of *ndufs4*, these mice demonstrate a delayed and initially less severe neurological phenotype compared to germline *ndufs4*^*−/−*^ mice and achieve an almost two-fold increase in lifespan, with *Vglut2-Cre;ndufs4*^*loxP/loxP*^ mice surviving to P90 with consistency but dying shortly thereafter.

**Figure 2:**
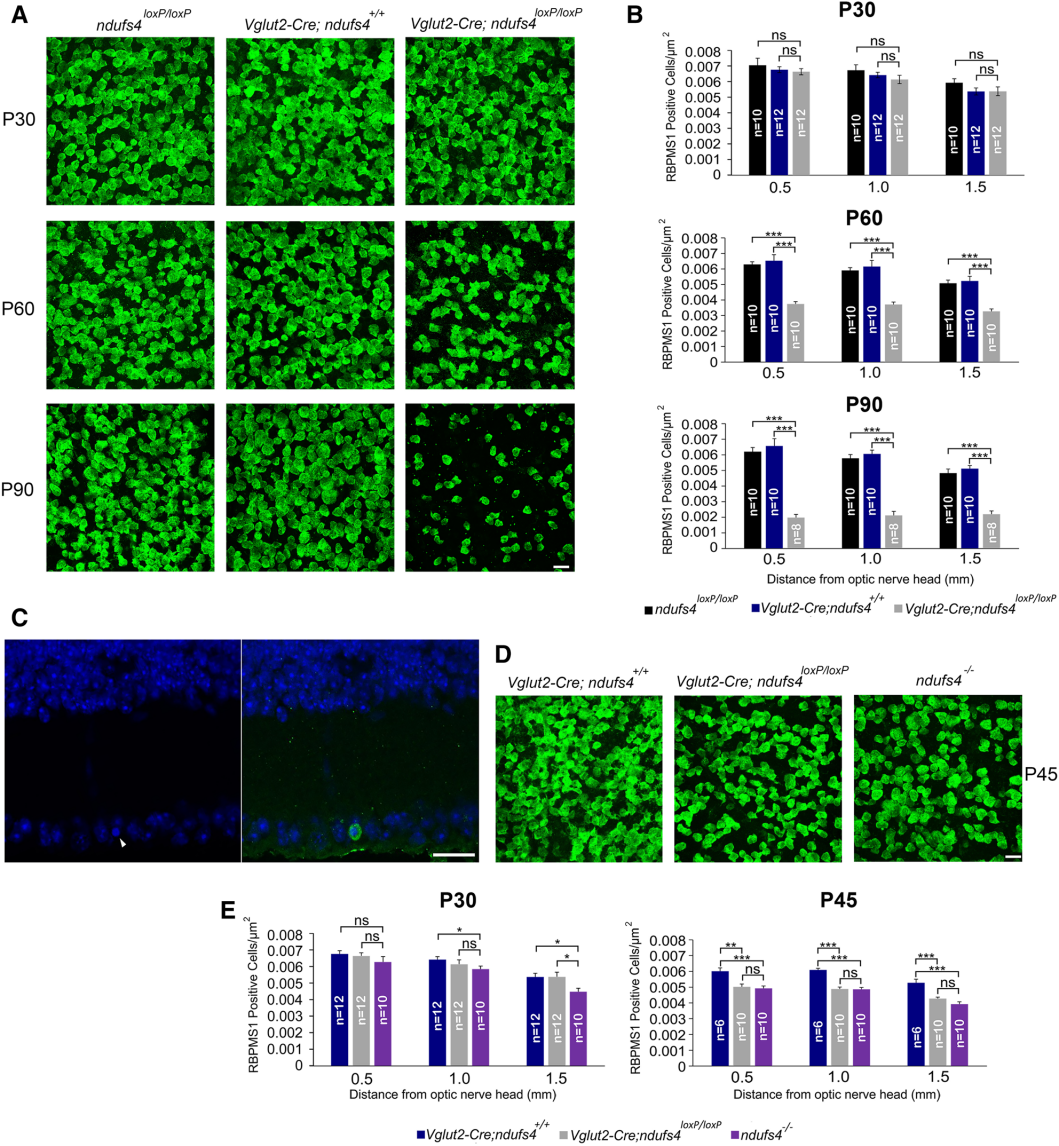
Retinal ganglion cell soma density declines rapidly after P30 in *Vglut2-Cre;ndufs4*^*loxP/loxP*^ retinas. (**A**) Retinal flat mounts from *ndufs4*^*loxP/loxP*^, *Vglut2-Cre;ndufs4*^+*/*+^*,* and *Vglut2-Cre;ndufs4*^*loxP/loxP*^ mice were immunolabeled with RBPMS1 at P30, P60, and P90. (**B**) Bar graphs comparing RGC density between the indicated genotypes at distances of 0.5, 1.0, and 1.5 mm from the optic nerve head. (**C**) Retinal cross section from a P60 *Vglut2-Cre;ndufs4*^*loxP/loxP*^ mouse stained with DAPI and labeled for cleaved caspase 3. A cell with a pyknotic nucleus (arrowhead, left panel) exhibited positive cleaved caspase 3 signal (green, right panel). (**D**) Retinal flat mounts from P45 *Vglut2-Cre;ndufs4*^+*/*+^*, Vglut2-Cre;ndufs4*^*loxP/loxP*^ and germline *ndufs4*^*−/−*^ mice labeled for RBMPS1. (**E**) Bar graphs comparing RGC density between *Vglut2-Cre;ndufs4*^+*/*+^*, Vglut2-Cre;ndufs4*^*loxP/loxP*^ and germline *ndufs4*^*−/−*^ retinal flat mounts at P30 and P45. The bars for *Vglut2-Cre;ndufs4*^+*/*+^ and *Vglut2-Cre;ndufs4*^*loxP/loxP*^ at P30 are reproduced from panel **B**. All scale bars, 20 µm. Quantitative data depicted as mean ± SEM. (*p < 0.05; **p < 0.01; ***p < 0.0001; *ns*, not significant; n = number of retinas analyzed).

**Figure 3:**
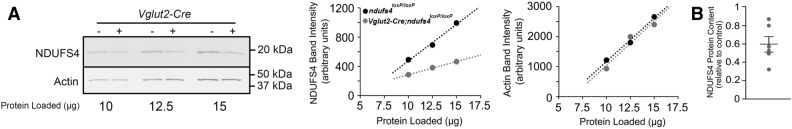
Assessment of NDUFS4 protein expression in mouse optic nerves. (**A**) Representative Western blot comparing the expression of NDUFS4 and actin in optic nerve lysates from *ndufs4*^*loxP/loxP*^ and *Vglut2-Cre; ndufs4*^*loxP/loxP*^ mice at P30. Total protein content of lysate loaded into each lane is indicated below, and the presence or absence of the *Vglut2-Cre* transgene is indicated above each lane. The graphs to the right depict NDUFS4 and actin band intensities plotted as a function of total protein loaded, demonstrating that the intensities are within a linear range for both proteins. (**B**) Quantification of NDUFS4 protein content in *Vglut2-Cre;ndufs4*^*loxP/loxP*^ optic nerve lysates relative to *ndufs4*^*loxP/loxP*^ littermates. NDUFS4 band intensity was normalized to actin band intensity for each sample, with 12.5 µg of total protein loaded per sample. n = 6 mice per genotype. Data are presented as mean ± SEM. See Supplementary Fig. S2 online for the uncropped blot from panel A.

The longer lifespan of the *Vglut2-Cre;ndufs4*^*loxP/loxP*^ mice afforded the opportunity to assess RGC degeneration over a three-month period. For this purpose, histological analysis of *Vglut2-Cre;ndufs4*^*loxP/loxP*^ retinas and optic nerves was compared to two different control genotypes, *ndufs4*^*loxP/loxP*^ and *Vglut2-Cre;nduf4*^+*/*+^, the latter serving to control for any potentially deleterious effect of the expression of Cre recombinase within RGCs. At P30, *Vglut2-Cre;ndufs4*^*loxP/loxP*^ mice demonstrated normal density of RGC somas in retinal flat mounts, consistent with the normal retinal development previously described in germline *ndufs4*^*−/−*^ mice^23^ (Fig. 2A,B). To determine whether *ndufs4* was successfully deleted in RGCs, we performed Western blot analysis to quantify the abundance of the protein product at P30. Because RGCs comprise < 1% of the total retinal cell population^30^, a reduction of NDUFS4 protein expression within RGCs would not be detectable in an analysis of whole retinas from *Vglut2-Cre;ndufs4*^*loxP/loxP*^ mice. Therefore, the analysis was performed on lysates from optic nerves, of which RGC axons are the dominant component (Fig. 3). At P30, the NDUFS4 protein content in *Vglut2-Cre;ndufs4*^*loxP/loxP*^ optic nerve tissue was reduced by variable levels compared to control *ndufs4*^*loxP/loxP*^ littermates, with a mean reduction of 40% [95% confidence interval for residual NDUFS4: (0.45, 0.75)]. Given that oligodendrocytes, astrocytes, and endothelial cells are also resident to the optic nerve^31^, our observation was consistent with a robust decrease in NDUFS4 content within RGC axons by P30. Because RGC density is normal at this age, the observed reduction in NDUFS4 protein content is not attributable simply to RGC degeneration.

Histological analysis at later time points revealed substantial degeneration of RGCs by P60, with a roughly 40% decrease in RGC density occurring at proximal, intermediate and distal locations from the optic nerve head (Fig. 2A,B). This was observed in both male and female mice. Moreover, a profound decline in the RGC population was observed at P90, with roughly two-thirds of RGCs lost at this final time point. Theoretically, the reduction of RGC somas in *Vglut2-Cre;ndufs4*^*loxP/loxP*^ retinas could simply reflect down-regulation of the cell marker RBPMS1, rather than RGC degeneration. Therefore, we quantified total nuclei in the innermost retinal layers (reflecting a mixture of RGCs, amacrine cells, and astrocytes) and confirmed a reduction of nuclei in P60 *Vglut2-Cre;ndufs4*^*loxP/loxP*^ retinas (Supplementary Fig. S1 online). Moreover, as shown in Fig. 2C, we observed occasional cells in the ganglion cell layer of *Vglut2-Cre;ndufs4*^*loxP/loxP*^ retinas which stained for cleaved caspase 3 (0.75 cells per retinal cross section, compared to 0.10 cells per cross section in *Vglut2-Cre;ndufs4*^+*/*+^ and 0.05 per cross section in *ndufs4*^*loxP/loxP*^ controls; p < 0.01 for both), indicative of a higher rate of apoptosis. There was no significant difference in RGC density between *ndufs4*^*loxP/loxP*^ and *Vglut2-Cre;nduf4*^+*/*+^ controls at any distance from the optic nerve head for any of the time points, indicating that the degeneration of RGCs in *Vglut2-Cre;ndufs4*^*loxP/loxP*^ mice was a result of the inactivation of *ndufs4* and not related to Cre toxicity (Fig. 2B).

The high specificity of *Vglut2*-driven Cre expression in RGCs suggests that RGC degeneration observed in *Vglut2-Cre;ndufs4*^*loxP/loxP*^ mice is a cell-autonomous process. To further explore this point, we directly compared the rate of RGC loss in *Vglut2-Cre;ndufs4*^*loxP/loxP*^ mice to that in germline *ndufs4*^*−/−*^ mice (Fig. 2D,E). At P30, RGC density was modestly but significantly decreased in *ndufs4*^*−/−*^ mice compared to *Vglut2-Cre;ndufs4*^*loxP/loxP*^ mice in the peripheral retina but not in more proximal regions. At P45, both *ndufs4*^*−/−*^ and *Vglut2-Cre;ndufs4*^*loxP/loxP*^ retinas demonstrated a 15–20% decrease in RGC density compared to control; there was no significant difference between *ndufs4*^*−/−*^ and *Vglut2-Cre;ndufs4*^*loxP/loxP*^ RGC densities at any of the distances from the optic nerve head at this time point. Later time points could not be assessed for the *ndufs4*^*−/−*^ mouse due to its shortened lifespan. Taken together, it would appear that mitochondrial dysfunction in other cell types has little, if any, impact on the early stages of degeneration of RGCs lacking *ndufs4*.

**Figure 4:**
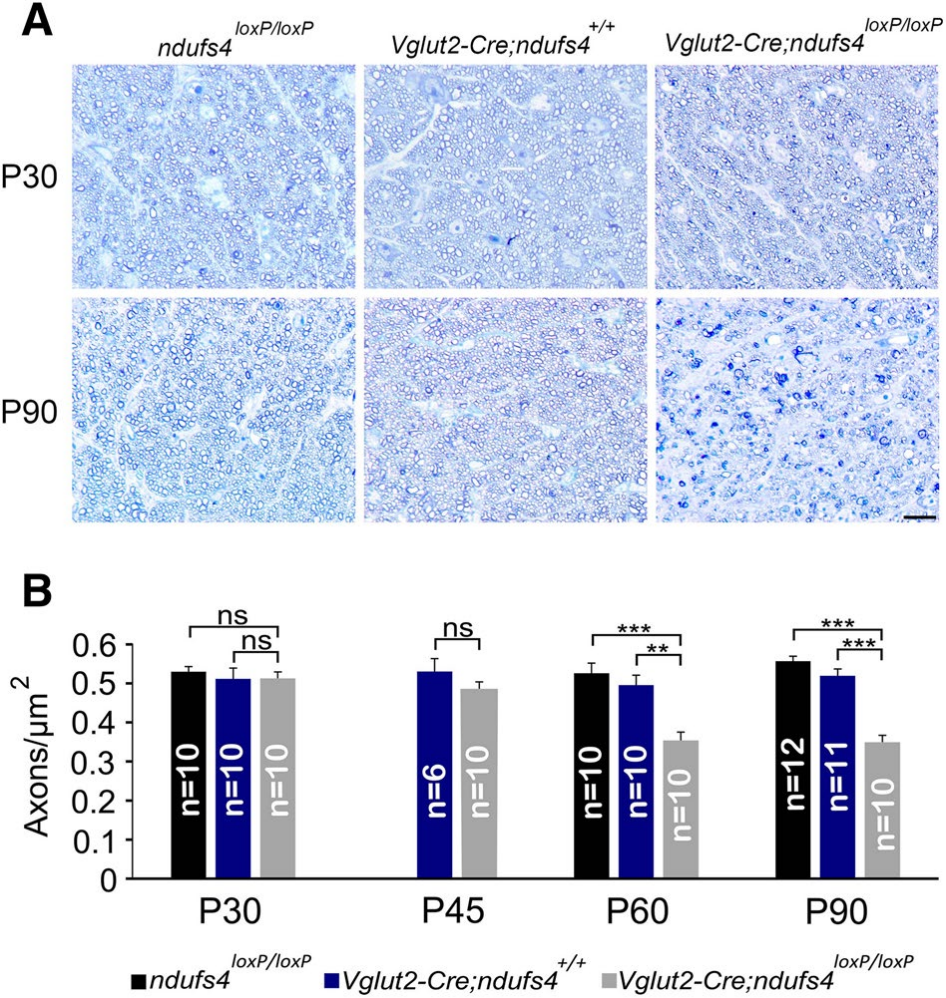
Optic nerve axon density declines in *Vglut2-Cre;ndufs4*^*loxP/loxP*^ mice by P60. (**A**) Representative light microscopy images of *ndufs4*^*loxP/loxP*^, *Vglut2-Cre;ndufs4*^+*/*+^*,* and *Vglut2-Cre;ndufs4*^*loxP/loxP*^ optic nerve cross-sections stained with methylene blue at P30 and P90. Scale bar, 20 μm. (**B**) Bar graphs comparing axon density between the indicated genotypes at the proximal portion of the optic nerve. Bars depict mean ± SEM; n = number of optic nerves per genotype; ** p < 0.001; ***, p < 0.0001; ns, not significant. Image acquisition and axon quantification performed with AxioVision SE64 Rel. 4.9.1 software.

Optic nerve cross-sections were also analyzed in order to quantify the density of RGC axons as a measure of optic atrophy. Consistent with our observations of RGC soma densities in retinal flat mounts, we found no difference in RGC axon densities between *Vglut2-Cre;ndufs4*^*loxP/loxP*^ optic nerves and the controls at P30 (Fig. 4A,B). By P60, *Vglut2-Cre;ndufs4*^*loxP/loxP*^ optic nerves exhibited ~ 33% reduction in axon density. Surprisingly, no further reduction was observed at P90, despite additional loss of RGC somas in retinal flat mounts at this time point. We suspected that this reflected a latency in retrobulbar axon loss, as the primary stress to RGCs in mitochondrial optic neuropathies is believed to occur at the level of the retina^24^. Supporting this explanation is our observation that at P45, RGC axon density was not significantly different in *Vglut2-Cre;ndufs4*^*loxP/loxP*^ optic nerves compared to control, despite RGC soma degeneration already occurring in the retina (compare Figs. 4B and 2E).

**Figure 5:**
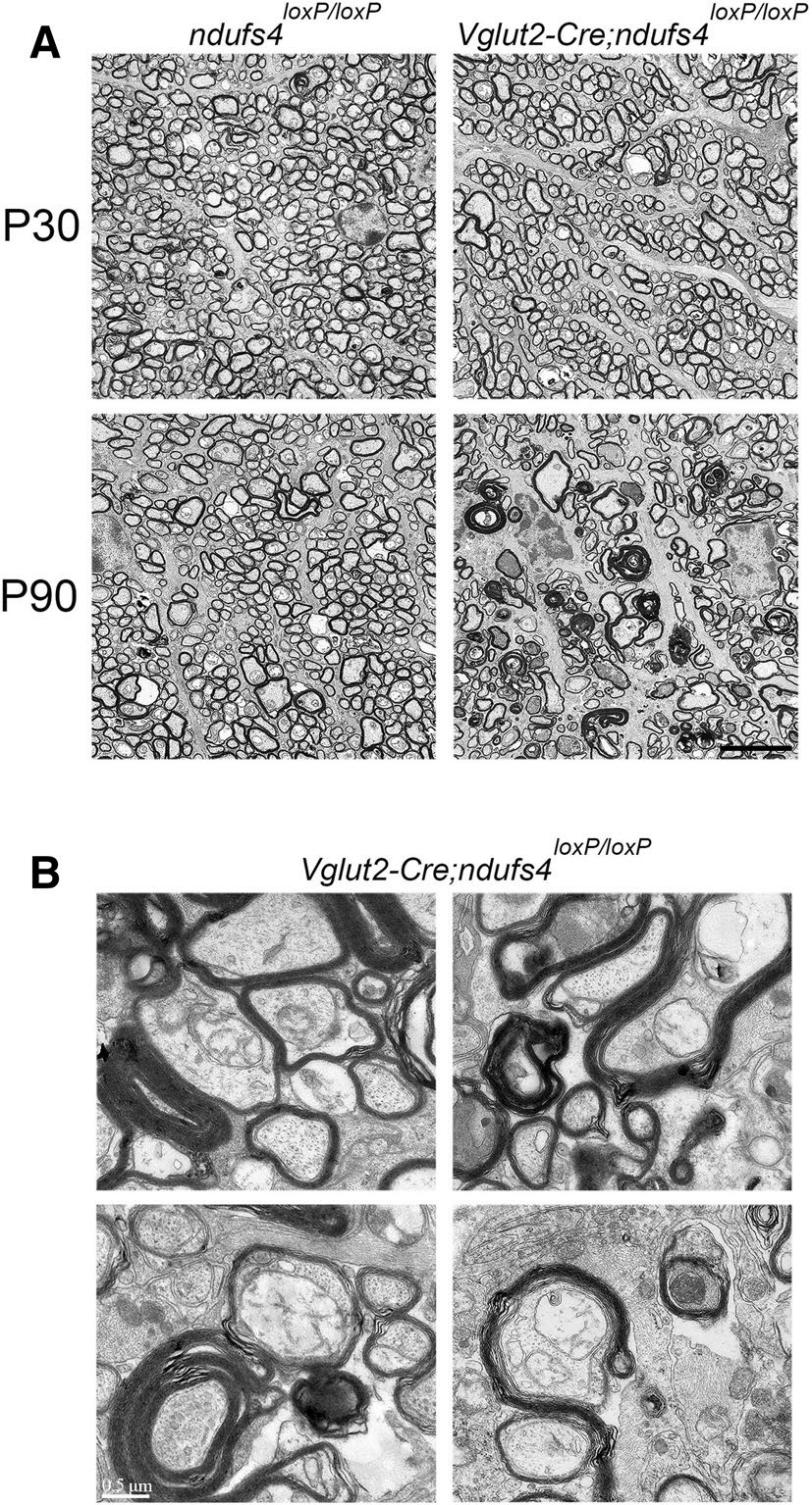
Electron microscopy of proximal optic nerve cross-sections. (**A**) Representative electron micrographs of *ndufs4*^*loxP/loxP*^ and *Vglut2-Cre;ndufs4*^*loxP/loxP*^ optic nerve cross-sections at P30 and P90, demonstrating axon loss and abnormal myelin figures at the latter time point in the *Vglut2-Cre;ndufs4*^*loxP/loxP*^ optic nerve. Magnification 5000×; scale bar, 5 μm. (**B**) Abnormal myelination patterns observed in P90 *Vglut2-Cre; ndufs4*^*loxP/loxP*^ optic nerve cross-sections, such as thickened myelin sheaths (upper left panel), redundant myelination (upper right, lower left) and incomplete axon enclosure (lower left and right). Magnification 40,000×; scale bar, 0.5 μm.

Evaluation of optic nerve cross-sections by electron microscopy confirmed normal morphology at P30 but lower RGC axon density and abnormal myelination patterns in P90 *Vglut2-Cre;ndufs4*^*loxP/loxP*^ optic nerves (Fig. 5A). Consistent with observations made in an LHON mouse model expressing mutant human ND4^19^, myelin sheaths were frequently thickened or even frankly redundant, with two or three discrete sheaths sometimes surrounding single axons (Fig. 5B). Some of the redundant myelin sheaths incompletely surrounded the axons, possibly representing residual myelin that originally enclosed axons having already undergone degeneration.

### In vivo electroretinography responses in Vglut2-Cre;ndufs4^loxP/loxP^ mice

**Figure 6:**
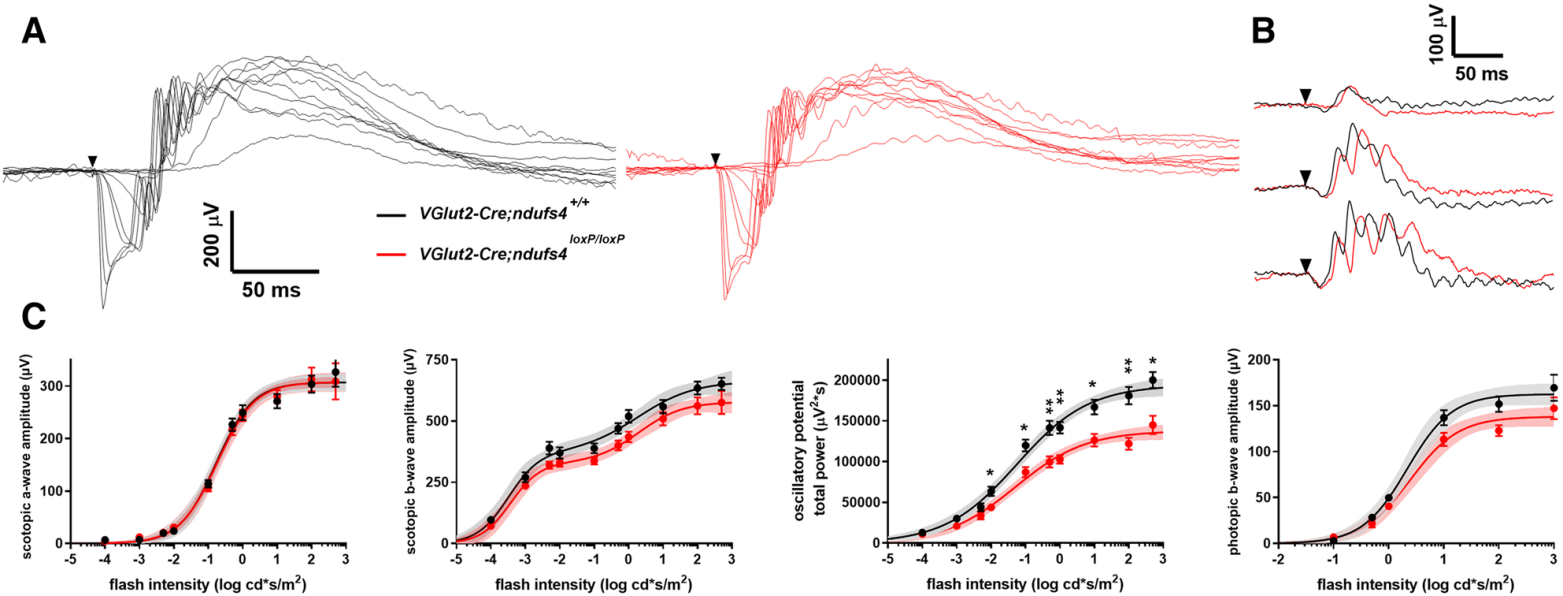
Flash electroretinography recordings in *Vglut2-Cre;ndufs4*^*loxP/loxP*^ mice. (**A**) Representative traces from ERG recordings of dark-adapted P45 *Vglut2-Cre;ndufs4*^+*/*+^ (black) and *Vglut2-Cre;ndufs4*^*loxP/loxP*^ mice (red) with stimulus intensities ranging from 0.0001 to 500 cd*s/m^2^. (**B**) Representative photopic light responses from the same mice, recorded at stimulus intensities of 1, 10, and 100 cd*s/m^2^ (from top to bottom) under rod-saturating background illumination of 30 cd/m^2^. (**C**) ERG responses of the *Vglut2-Cre;ndufs4*^+*/*+^ (black) and *Vglut2-Cre;ndufs4*^*loxP/loxP*^ mice (red) are plotted as a function of flash intensity and fit using a double or single hyperbolic function. Depicted from left to right are scotopic a-wave amplitude; scotopic b-wave amplitude; oscillatory potential total power; and photopic b-wave amplitude. Ten eyes of 5 *Vglut2-Cre;ndufs4*^+*/*+^ (black) and 12 eyes of 6 *Vglut2-Cre;ndufs4*^*loxP/loxP*^ mice were analyzed. Data are presented as mean ± SEM. Two-tailed *t*-tests comparing responses for each genotype did not reach a significance level of p = 0.05 for any flash intensity, except for oscillatory potentials obtained at the seven highest flash intensities; *, p < 0.05; **, p < 0.01.

To assess light signaling function at various levels of the retina, we performed electroretinography (ERG) recordings on dark-adapted *Vglut2-Cre;ndufs4*^*loxP/loxP*^ mice and *Vglut2-Cre;nduf4*^+*/*+^ control littermates. Previous ERG analyses of germline *ndufs4*^*−/−*^ mice revealed reduced a- and b-wave amplitudes, consistent with a decreased light response at the level of photoreceptor cells^22,23,32^. However, since we observed *Vglut2*-driven Cre expression in only a minority of cones and in no rods or bipolar cells, little effect on these parameters was expected in *Vglut2-Cre;ndufs4*^*loxP/loxP*^ mice. Scotopic (Fig. 6A) and photopic (Fig. 6B) ERG recordings were obtained at P45, an age at which a maximal ~ 50% reduction of a- and b-wave amplitudes has already occurred in germline *ndufs4*^*−/−*^ mice^32^. As shown in Fig. 6C, a-wave amplitudes in *Vglut2-Cre;ndufs4*^*loxP/loxP*^ mice were indistinguishable from the *Vglut2-Cre;nduf4*^+*/*+^ controls. b-wave amplitudes were slightly decreased in both scotopic (~ 15% reduction) and photopic (~ 20%) recordings, although these reductions were not statistically significant at any stimulus intensity. In contrast, oscillatory potentials were significantly decreased by ~ 30% in *Vglut2-Cre;ndufs4*^*loxP/loxP*^ mice compared to *Vglut2-Cre;nduf4*^+*/*+^ littermates (Fig. 6C), reflecting impairment of feedback signaling in inner retinal neurons, including RGCs^33^.

### *Vglut2-Cre;ndufs4*^*loxP/loxP*^ retinas exhibit signs of neuroinflammation

**Figure 7:**
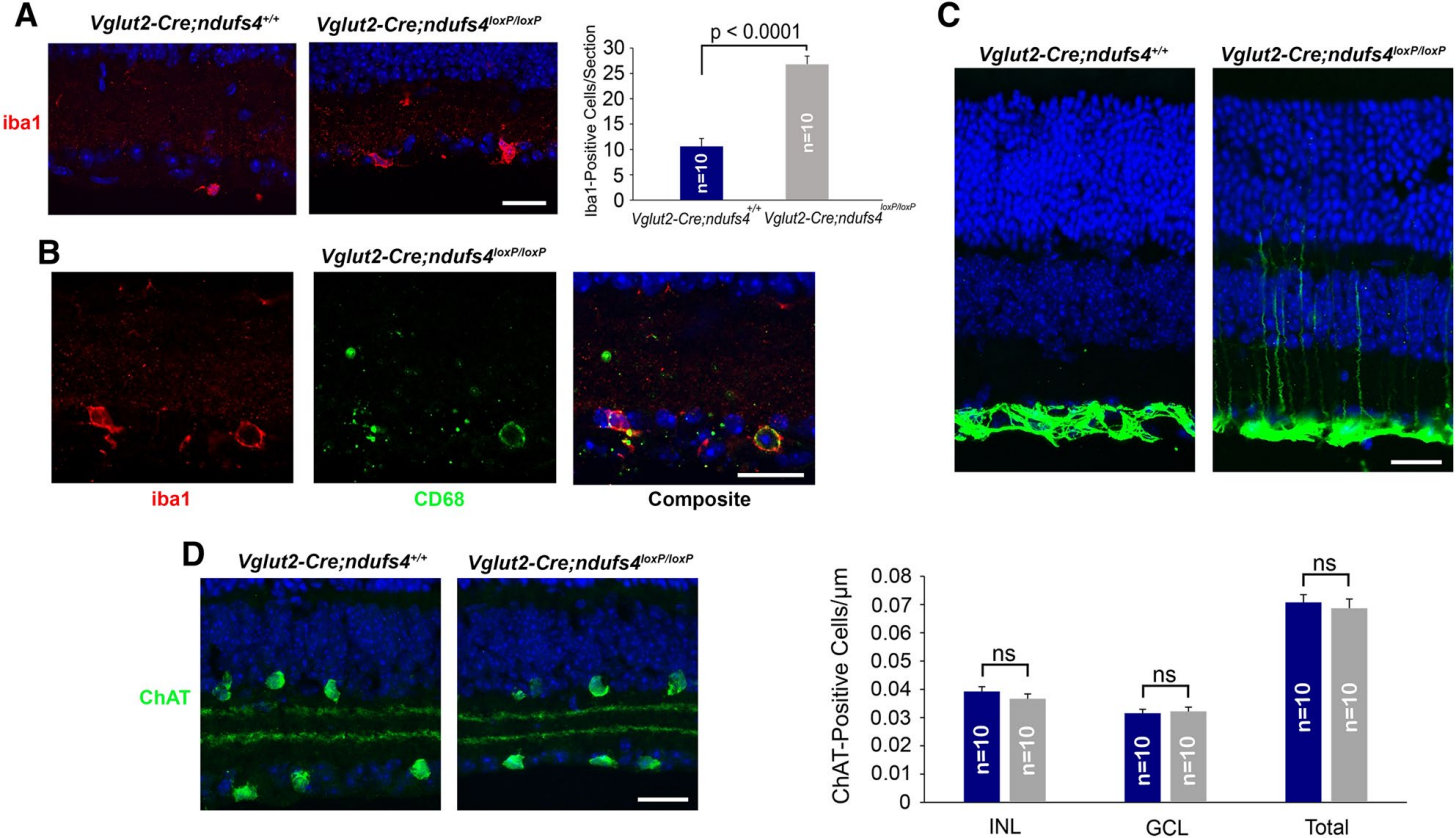
Neuroinflammation in *Vglut2-Cre;ndufs4*^*loxP/loxP*^ retinas. (**A**) Iba1 immunolabeling of mononuclear cells in cross sections of P60 *Vglut2-Cre;ndufs4*^+*/*+^ and *Vglut2-Cre;ndufs4*^*loxP/loxP*^ retinas. Bar graph to the right depicts mean number of iba1-positive nuclei per retinal section. (**B**) Representative image of *Vglut2-Cre;ndufs4*^*loxP/loxP*^ retina with activated myeloid cells co-labeled for iba1 and CD68. (**C**) GFAP immunolabeling of *Vglut2-Cre;ndufs4*^+*/*+^ and *Vglut2-Cre;ndufs4*^*loxP/loxP*^ retinal cross sections. (**D**) Immunolabeling of starburst amacrine cells with ChAT. Bar graph to the right compares the mean number of ChAT-positive cells in the INL, GCL, and combined for *Vglut2-Cre;ndufs4*^+*/*+^ (blue) and *Vglut2-Cre;ndufs4*^*loxP/loxP*^ retinas (gray). For both bar graphs, n = number of retinas analyzed, with 3 sections averaged per retina. Data are presented as mean ± SEM. *ns* not significant, *INL* inner nuclear layer, *GCL* ganglion cell layer. All scale bars, 20 μm.

It has been previously reported that RGC degeneration in the germline *ndufs4*^*−/−*^ mouse is accompanied by infiltration of activated myeloid cells into the inner plexiform and ganglion cell layers^23^. To determine whether neuroinflammation also occurs in the setting of *Vglut2*-driven conditional deletion of *ndufs4* from RGCs, we quantified the abundance of iba1-positive mononuclear cells on cryosections obtained from P60 *Vglut2-Cre;ndufs4*^*loxP/loxP*^ mice and *Vglut2-Cre;nduf4*^+*/*+^ control littermates. Indeed, a greater than two-fold increase in iba1-positive cells was observed within the inner retina of *Vglut2-Cre;ndufs4*^*loxP/loxP*^ mice (Fig. 7A), and 96% of these cells co-labeled for CD68 (Fig. 7B), indicative of an active, phagocytic state. These cells may represent resident microglia or recruited myeloid cells, as iba1 cannot distinguish between these cell populations. Furthermore, whereas control retinas exhibited normal confinement of the intermediate filament glial fibrillary acidic protein (GFAP) to astrocytes and Müller cell end-feet in the ganglion cell layer, *Vglut2-Cre;ndufs4*^*loxP/loxP*^ retinas demonstrated up-regulated GFAP localizing to Müller cell radial processes, consistent with reactive gliosis (Fig. 7C). Per 100-µm segment of inner plexiform layer in retinal cross sections, 8.6 ± 3.7 (mean ± SEM) GFAP-positive radial processes were observed in *Vglut2-Cre;ndufs4*^*loxP/loxP*^ retinas, while none were observed in *Vglut2-Cre;nduf4*^+*/*+^ controls (p = 0.02). However, unlike what we and others have observed in germline *ndufs4*^*−/−*^ retinas^23,32^, there was no associated decrease in starburst amacrine cell abundance in the ganglion cell layer of *Vglut2-Cre;ndufs4*^*loxP/loxP*^ retinas (Fig. 7D), again supporting the notion that the observed RGC death is not dependent on inflammation induced by the death of neighboring cell types.

## Discussion

In this study, we report that conditional deletion of *ndufs4* in *Vglut2*-expressing neurons results in progressive loss of RGC somas and axons, with associated neuroinflammation of the inner retina. This phenomenon is entirely consistent with the phenotype observed at the end of the short lifespan of germline *ndufs4*^*−/−*^ mice^23^, but the prolonged survival of *Vglut2-Cre;ndufs4*^*loxP/loxP*^ mice allows this pathology to progress substantially further.

By using a tdTomato reporter to assess the extent of *Vglut2*-driven Cre expression in other retinal neurons, we have addressed the lingering question of whether or not RGC degeneration in the setting of complex I deficiency is a cell-autonomous process. Prior characterizations of the germline *ndufs4*^*−/−*^ mouse indicated that the onset of RGC death is preceded by death of starburst amacrine cells^23^ and potentially rod bipolar cells [^20^, but see^32^]. This raised the possibility that RGC degeneration was secondary to dysfunction of cells synapsing on RGCs and/or to the recruitment of inflammatory cells to the inner retina in response to death of neighboring cell types. However, *Vglut2*-driven Cre is not expressed in starburst amacrine cells or in rod bipolar cells in our model, nor did we observe any dropout of starburst amacrine cells by P60, when RGC degeneration was well underway. This, combined with the fact that RGC degeneration commences with similar kinetics in *Vglut2-Cre;ndufs4*^*loxP/loxP*^ mice as in *ndufs4*^*−/−*^ mice, suggests that intrinsic complex I dysfunction within RGCs is sufficient to produce optic atrophy. Future experiments assessing Cre expression and degeneration of post-synaptic neurons in the superior colliculus could rule out the possibility of retrograde trans-synaptic degeneration as a contributing mechanism to the RGC loss observed in this model.

In contrast to germline *ndufs4*^*−/−*^ mice, which exhibit ~ 50% reduction of a- and b-wave amplitudes^32^, preganglionic retinal signaling was minimally affected in ERG recordings from *Vglut2-Cre;ndufs4*^*loxP/loxP*^ mice. While the scotopic a-wave was completely unaffected (indicative of normal phototransduction), there was a trend of modestly reduced b-wave amplitudes in both scotopic and photopic recordings. We can only speculate on the reason for this. Reduced b-waves would typically be thought to reflect abnormal synaptic transmission between photoreceptors and bipolar cells or abnormal signaling intrinsic to bipolar cells. However, while a subset of cone photoreceptors exhibited Cre-dependent tdTomato expression, no rod photoreceptors or bipolar cells expressed Cre in *Vglut2-Cre;ndufs4*^*loxP/loxP*^ mice and would therefore be expected to be physiologically normal. It is conceivable that a mild reduction in b-wave amplitudes could be an indirect effect of RGC pathology and/or inner retinal inflammation, as animal models of traumatic or inflammatory optic neuropathy have exhibited variable effects on outer retinal signaling properties^34,35^. The reduction in oscillatory potentials observed in ERG recordings of the conditional knockout mice is consistent with abnormal RGC function, as seen previously in mouse glaucoma models^33,36^. However, oscillatory potentials derive from complicated inhibitory feedback pathways involving multiple inner retinal cell types, and we cannot rule out that our observations relate more to the presence of inner retinal inflammation than directly to RGC dysfunction/degeneration. Future electrophysiological analysis of *Vglut2-Cre;ndufs4*^*loxP/loxP*^ mice with pattern ERGs may better isolate the impact of complex I deficiency on RGC function and potentially allow pathology to be detected prior to the onset of overt degeneration.

Efficient testing of potential pharmacotherapies for mitochondrial optic neuropathies depends on the availability of an animal model exhibiting reliable and rapidly progressive RGC degeneration with a clinically relevant underlying pathobiology. We believe that the *Vglut2-Cre;ndufs4*^*loxP/loxP*^ transgenic mouse line satisfies these criteria and promises to be a valuable preclinical model for optic neuropathies directly related to complex I dysfunction, such as LHON. We show that RGCs with inactivated *ndufs4* begin to die at ~ P45, with degeneration becoming profound by P90. Similar to prior characterization of the germline *ndufs4*^*−/−*^ mouse^23^, *Vglut2-Cre;ndufs4*^*loxP/loxP*^ RGCs develop in normal abundance and show no axonal pathology at the time the mice initially reach maturity. The absence of congenital anomalies is an important feature of the ocular phenotype in these mice, as LHON also is associated with structurally and functionally normal optic nerves until the onset of overt vision loss, typically after patients reach puberty.

Although the shortened lifespan of *Vglut2-Cre;ndufs4*^*loxP/loxP*^ mice of ~ 90 days limits the duration of experiments possible in this model, the nearly two-fold increase in lifespan compared to *ndufs4*^*−/−*^ mice is paramount, as most of the RGC degeneration occurs outside of the lifespan of the germline knockout mouse. The fact that *Vglut2-Cre;ndufs4*^*loxP/loxP*^ mice already exhibit robust RGC degeneration at P60 means that the protective effect of any potential therapeutic strategy could be determined reliably at P60 and then assessed for durability over an additional month. Importantly, given that most human patients with LHON are not diagnosed until they have already developed some degree of vision loss in both eyes, this model will also provide the opportunity to test the ability of therapies to halt the progression of optic atrophy when administered after RGC degeneration has already commenced.

In addition to being a superior model to the germline *ndufs4* knockout, the *Vglut2-Cre;ndufs4*^*loxP/loxP*^ mouse line has several potential advantages over other existing mouse models of complex I-associated optic neuropathy. One non-genetic strategy has been to expose animals to the toxin rotenone, a potent inhibitor of complex I. To reduce systemic toxicities, this may be administered by injection into the vitreous cavity or the superior colliculus to achieve complex I inhibition within RGCs^37,38^. Rotenone toxicity rapidly and efficiently induces optic atrophy (producing RGC death as soon as 24 h after treatment in the case of intravitreal injections). This rapidly-induced pathology may phenocopy the acute vision loss experienced by some LHON patients better than our model does. However, rotenone injections require that an invasive procedure be performed on each animal and introduce a sudden metabolic insult to the RGCs that does not mimic the chronic metabolic stress experienced by these cells in heritable mitochondrial diseases. Another popular somatic approach is to generate an LHON-like phenotype through allotopic expression of complex I mutations in RGCs via in vivo electroporation or transduction by adeno-associated virus (AAV)^39,40^. However, these manipulations are not heritable (again requiring intravitreal injection of each animal, with the accompanying risk of iatrogenic retinal damage), and the efficiency of mitochondrial import of the mutant proteins and subsequent incorporation into endogenous respiratory complexes is potentially problematic, as protein aggregation in the cytosol could potentially be harmful to RGCs, irrespective of the effect on complex I function^5,41^.

The two existing heritable mouse models of mitochondrial optic neuropathy represent the most relevant comparisons for the present model. Lin et al*.* engineered a mouse line that is homoplasmic for a mtDNA G13997A point mutation resulting in a P25L amino acid substitution in the complex I subunit ND6^18^. This mouse holds the advantage of bearing a *bona fide* mitochondrial disease mutation seen in a family with Leigh syndrome, unlike the deletion of exon 2 in *ndufs4*, which has not been described in humans. However, the slow progression of optic atrophy in G13997A mice impedes large-scale testing of pharmacotherapies or gene therapies in this model, with no RGC axonal loss observed at 14 months and only ~ 25% by 24 months^18^ (compared to 33% by P60 in *Vglut2-Cre;ndufs4*^*loxP/loxP*^ mice). More recently, Yu et al*.* utilized a mitochondria-targeted AAV to deliver an episomally-expressed human ND4 mutation that is transmitted through the germline and results in mice that are functionally heteroplasmic for the mutation^19^. This model demonstrates profound RGC soma and axon loss by 22 months. Although histological assessment at younger ages has not been reported, pattern ERG recordings from this mouse suggest that the onset of RGC degeneration likely takes place at least two months later than in *Vglut2-Cre;ndufs4*^*loxP/loxP*^ mice. One final advantage is that, unlike a mouse model with a mitochondrial inheritance pattern, the *Vglut2-Cre;ndufs4*^*loxP/loxP*^ mouse line described here may be used to generate control littermates for experiments (e.g. half of the progeny from the cross of a *Vglut2-Cre;ndufs4*^*loxP/loxP*^ male to a *ndufs4*^*loxP/loxP*^ female will be *ndufs4*^*loxP/loxP*^ mice that do not develop optic atrophy). While we acknowledge that *ndufs4* mutations have not been directly implicated in LHON, a mutation-independent therapeutic strategy that is protective of RGCs in the setting of *ndufs4* inactivation would seem to have a high likelihood of efficacy in treating the much less severe complex I dysfunction seen in LHON patients.

## Methods

### Animals

All animal procedures were performed in accordance with the NIH Guide for the Care and Use of Laboratory Animals. The Institutional Animal Care and Use Committee of Duke University approved all experimental protocols. All mice were on a C57BL/6 genetic background and purchased from the Jackson Laboratory (Bar Harbor, ME). Transgenic mice homozygous for *Vglut2-Cre* (stock number 028863)^42^ were crossed with *Ai9* mice (stock number 007909)^43^ to achieve Cre-dependent tdTomato reporter expression. Homozygous *Vglut2-Cre* mice were also crossed with transgenic *ndufs4*^*loxP/loxP*^ mice in which exon 2 of *ndufs4* is *floxed* (stock number 026963)^22^ in order to generate *Vglut2-Cre;ndufs4*^*loxP/*+^ progeny. The F_1_ mice were then crossed to produce *Vglut2-Cre;ndufs4*^*loxP/loxP*^ conditional knockout mice, as well as *Vglut2-Cre;ndufs4*^+*/*+^ and *ndufs4*^*loxP/loxP*^ control littermates. Finally, heterozygous mice carrying a germline deletion of exon 2 of *ndufs4* (stock number 027058)^22^ were crossed to generate homozygous *ndufs4*^*−/−*^ progeny. Animals were reared under a normal day/night cycle and all experiments were performed during the day.

### Antibodies

The following antibodies were used for immunofluorescence experiments: rabbit polyclonal anti-RBPMS (1:500; Novus, NBP2-20112, Lot#130–96), rabbit polyclonal anti-cone arrestin (1:500, EMD Millipore, AB15282, Lot#3156749), mouse monoclonal anti-calbindin D28K (D-4) (1:500, Santa Cruz Biotechnology, sc-365360, Lot#3156749), mouse monoclonal anti-PKC-α H-7 (1:100; Santa Cruz, sc-8393, Lot#I1306), goat polyclonal anti-choline acetyltransferase (1:100; Millipore, AB144P, Lot#3169862), rabbit monoclonal anti-mCherry (1:500, Abcam, EPR20579, Lot#ab213511), rabbit polyclonal anti-cleaved caspase 3 (Asp175) (1:200, Cell Signaling, 9661 T, Lot#45), rabbit polyclonal anti-iba1 (1:1000; Fujifilm Wako Pure Chemicals Corp., 019–19741), rat monoclonal anti-CD68 FA-11 (1:200; BioLegend, 137002), and rabbit polyclonal anti-GFAP (D1F4Q) XP (1:200; Cell Signaling Technology, #12389). The following antibodies were used for Western blot analysis of optic nerve lysates: mouse monoclonal anti-NDUFS4 1-E-4 (1:200; Santa Cruz, sc-100567, Lot#A2918) and mouse monoclonal anti-β-actin (1:1000; Santa Cruz, sc-47778, Lot#D0615). Secondary antibodies against the appropriate species conjugated to Alexa Fluor 488, Alexa Fluor 568 (immunofluorescence experiments, 1:500 dilution) or Alexa Fluor 680 (Western blot experiments, 1:20,000 dilution) were purchased from Invitrogen. Cell nuclei were stained using DAPI (Sigma Aldrich).

### Electroretinography

ERG recordings were performed on live mice using the Espion E2 system with a ColorDome Ganzfeld stimulator (Diagnosys LLC, Littleton, MA) as described previously^44,45^. Mice were anesthetized via intraperitoneal injection of 100 mg/kg ketamine and 10 mg/kg xylazine following a 6-h dark adaptation. A mixture of 1% cyclopentolate-HCl and 2.5% phenylephrine was used to dilate the pupils. During recordings, eyes were lubricated with 1% carboxymethylcellulose sodium gel, and body temperature was maintained using a heated platform. Recordings were conducted simultaneously in both eyes using gold contact lens electrodes (Mayo Corp., Aichi, Japan), with stainless steel needle electrodes (Ocuscience, Henderson, NV) placed in the mouth (reference) and at the base of the tail (ground). ERG signals were sampled at 1 kHz and recorded with 0.15 Hz low-frequency and 500 Hz high-frequency cutoffs. Responses to flashes from 0.0001 to 500 cd*s/m^2^ with 10 to 1 trials averaged and inter-flash intervals of 5–180 s were recorded in the dark. Then, flashes from 0.1 to 1000 cd*s/m^2^ were recorded in the presence of rod-saturating light of 30 cd/m^2^ to isolate cone responses. Following the recordings, the mice were euthanized for histological experiments.

Data from the ERG recordings were analyzed using Matlab 2019a (MathWorks). Oscillatory potentials were removed from the signals by 55 Hz fast Fourier transform (FFT) low-pass frequency filtering of the early part of the response and 2nd-order butterworth filtering of the non-photoreceptor-driven signal. The amplitude of b-waves was calculated from baseline to the peak for dimmer flashes and from the bottom of the a-wave to the b-wave peak for brighter flashes, and the amplitude of the a-wave was calculated from baseline to trough. To calculate the total energy of the oscillatory potentials, a trapezoidal numerical integration was performed on a 65 to 300 bandpass FFT for each 5^th^-order butterworth filtered response^33^. *p*-values were calculated to determine the statistical significance between the response amplitudes of *Vglut2-Cre;ndufs4*^*loxP/loxP*^ and control mice at each flash intensity using two-tailed *t*-tests in GraphPad software.

### Histological techniques

For immunohistochemistry experiments, eyes were enucleated from euthanized mice and the anterior segments were removed to generate posterior eyecups, which were fixed for 1 h in 4% paraformaldehyde and then rinsed in phosphate-buffered saline solution (PBS). Retinal flatmounts were prepared by carefully dissecting retinas from the eyecups and blocking in 5% goat serum in PBS with 0.3% Triton X-100 for 2 h at room temperature, then incubating with anti-RBPMS primary antibody in block for 7 days at 4 °C. Retinas were washed and then incubated with donkey anti-rabbit Alexa Fluor 488 in block overnight at 4 °C. The retinas were then washed and placed on glass slides with the RGC layer facing up, and four radial cuts were made from the edge to the equator of each retina to achieve flattening prior to mounting. For nuclei counts, isolated fixed retinas were incubated in DAPI overnight prior to washing and mounting on slides.

Retinal cryosections were prepared by cryoprotecting fixed eyecups in 30% sucrose and then embedding them in optimal cutting temperature (OCT) medium (Tissue-Tek, Sakura Finetek). Retinal cross-sections, 14 μm in thickness (for iba1, CD68, and ChAT staining) or 20 μm in thickness (all others), were collected using a cryostat microtome (Microm HM 550, Thermo Fisher Scientific). Sections were rehydrated with PBS, blocked in 5% goat or donkey serum in PBS with 0.3% Triton X-100 for 2 h at room temperature, and then incubated in primary antibody in the same blocking solution overnight at 4 °C. Sections were washed and incubated with appropriate secondary antibody conjugated to Alexa Fluor 488 or Alexa Fluor 568 in block solution overnight at 4 °C. The sections were then washed prior to mounting.

All samples were mounted with Vectashield (Vector Laboratories, Burlingame, CA) under glass coverslips. Images were acquired using a Nikon Eclipse Ti2 inverted confocal microscope, a CFI Plan Fluor 60 × (oil) objective, and an A1 confocal scanner controlled by NIS-Elements software (Nikon, Tokyo, Japan). For quantification of retinal ganglion cell somas and inner retinal nuclei in retinal flatmounts, z-stacks through the retinal nerve fiber layer and ganglion cell layer of 45,000 µm^2^ in area were obtained in each quadrant at locations of 0.5, 1.0, and 1.5 mm from the optic nerve head. RBPMS1-positive RGC somas and DAPI-stained nuclei were manually counted using the Cell Counter plugin for Fiji^46,47^. Cell density was averaged among the four quadrants at each distance from the optic nerve head. For quantification of cells positive for calbindin, tdTomato, cleaved caspase 3, iba1, and CD68, retinal cross sections were imaged along their entire length, and the number of positive cells was quantified in 3–4 retinal sections per sample (taken through the optic nerve head), then averaged. For quantification of cells labeled for cone arrestin, ChAT, and GFAP, 45,000 µm^2^ images were acquired at a 500-µm distance to either side of the optic nerve head for 3 sections per sample. For cone arrestin, the overall number of positive cell bodies and number positive for tdTomato were counted. For ChAT, the average number of positive cells/µm was calculated separately for the inner nuclear layer and the ganglion cell layer. For GFAP, the number of positive radial processes present at the inner nuclear layer/inner plexiform layer junction was counted.

To assess RGC axons, mouse optic nerve specimens were processed and analyzed as previously described^48^. Briefly, euthanized mice were exsanguinated via transcardial perfusion with 80 mL of ice-cold PBS followed by fixation with 80 mL of 4% PFA. Optic nerves were dissected back to the optic chiasm and fixed in 2% paraformaldehyde and 2% glutaraldehyde overnight, then washed in PBS. Samples were embedded in the Embed-812 resin mixture and sectioned on an ultramicrotome (LKB Ultratome V; Leica, Paris, France) using a glass knife. Cross-sections of 0.27 µm thickness were stained with 1% methylene blue. Axon counts were obtained using the AxioSkop 2 Plus upright microscope and AxioVision SE64 Release 4.9.1 imaging software (Zeiss, Oberkochen, Germany; https://www.micro-shop.zeiss.com/en/us/system/axiovision+software/software+axiovision/axiovision+program/410130-0909-000#/). For each optic nerve, 4 images of 69.5 µm × 87.7 µm were obtained using a 100X (oil) objective. Image analysis consisted of automated RGB thresholding, as well as size and form factor exclusions, followed by manual exclusion of inappropriately identified axons. Approximately 40% of the total cross-sectional area of each optic nerve was sampled and counted. The final count was divided by the total area sampled to determine mean axon density per nerve.

The same mouse optic nerve specimens processed for light microscopy were thinly sectioned (60–80 nm) for transmission electron microscopy. Samples were collected on copper grids, counterstained with uranyl acetate and Sato’s lead, and then examined using an electron microscope (JEM-1400; JEOL, Tokyo, Japan) at 60 kV. Images were collected using a charge-coupled device camera (Orius; Gatan, Pleasanton, CA).

### Western blot for NDUFS4 quantification

The optic nerves of euthanized mice were dissected and then sonicated in lysis buffer [25 mM HEPES buffer, pH 7.4, 150 mM NaCl, 5 mM MgCl_2_, and protease inhibitors (Complete Mini, Roche, Indianapolis, IN) with 1% Triton X-100] and the protein concentration of each lysate was determined with a colorimetric assay (Bio-Rad). After mixing with SDS-PAGE sample buffer, optic nerve lysates from *ndufs4*^*loxP/loxP*^ and *Vglut2-Cre;ndufs4*^*loxP/loxP*^ mice were separated on 4–20% SDS-PAGE gels, transferred onto polyvinylidene fluoride (PVDF) membranes and blotted with the indicated primary antibodies overnight. Membranes were washed in 0.05% Tween X-20 and incubated with the appropriate secondary antibody for two hours at room temperature. The Odyssey CLx imaging system (LI-COR, Lincoln, NE) was used to image and quantify band intensities. 12.5 µg of total protein was loaded for each sample, as signal intensities for actin and NDUFS4 were both found to be within a linear range at this input.

### Experimental design and statistical analysis

All histological experiments and in vivo electrophysiological experiments were performed on *Vglut2-Cre;ndufs4*^*loxP/loxP*^ mice and littermate controls with both sexes represented. A minimum of 5 retinas or optic nerves were analyzed for each genotype at each time point for quantitative histological experiments. RGC soma and optic nerve axon densities were compared between genotypes using two-tailed *t-*tests on GraphPad Prism version 8.00 for Windows (GraphPad Software). Data are presented as mean ± SEM. All in vivo ERG recordings were performed at P45 (10 eyes of 5 *Vglut2-Cre;ndufs4*^*loxP/loxP*^ mice and 12 eyes of 6 *Vglut2-Cre;ndufs4*^+*/*+^ mice). The a-wave and b-wave amplitudes and oscillatory potential energy recorded at each flash intensity were compared using a two-tailed *t*-test.

## Supplementary information

Supplementary file1

## Data Availability

The data that support the findings of this study are available from the corresponding author on reasonable request.
