# Supplementary material for: Progressive optic atrophy in a retinal ganglion cell-specific mouse model of complex I deficiency
Source: Sci Rep. 2020 Oct 1;10:16326. doi: 10.1038/s41598-020-73353-0 (PMC7529752; doi:10.1038/s41598-020-73353-0)
Supplement: Supplementary file 1 — Supplementary file1 [file 41598_2020_73353_MOESM1_ESM.pdf]

# Supplementary Information

**TITLE**

Progressive Optic Atrophy in a Retinal Ganglion Cell-Specific Mouse Model of Complex I Deficiency

**AUTHORS**

Luyu Wang[1], Mikael Klingeborn[1], Amanda M. Travis[2], Ying Hao[1], Vadim Y. Arshavsky[1,3], Sidney M. Gospe, III[1,*]

[1] Department of Ophthalmology, Duke University School of Medicine, Durham, NC 27710, USA

[2] Department of Ophthalmology and Visual Sciences, University of Michigan, Ann Arbor, MI 48105, USA

[3] Department of Pharmacology & Cancer Biology, Duke University School of Medicine, Durham, NC 27710, USA

*Address correspondence and reprint requests to:

Sidney M. Gospe, III, MD, PhD
Department of Ophthalmology
Box 3712 Med Center
Duke University
2351 Erwin Road
Durham, NC 27710
Tel: 919-681-9191
Fax: 919-684-0547
E-mail: sid.gospe@duke.edu

**Figure S1**

**Figure S2**

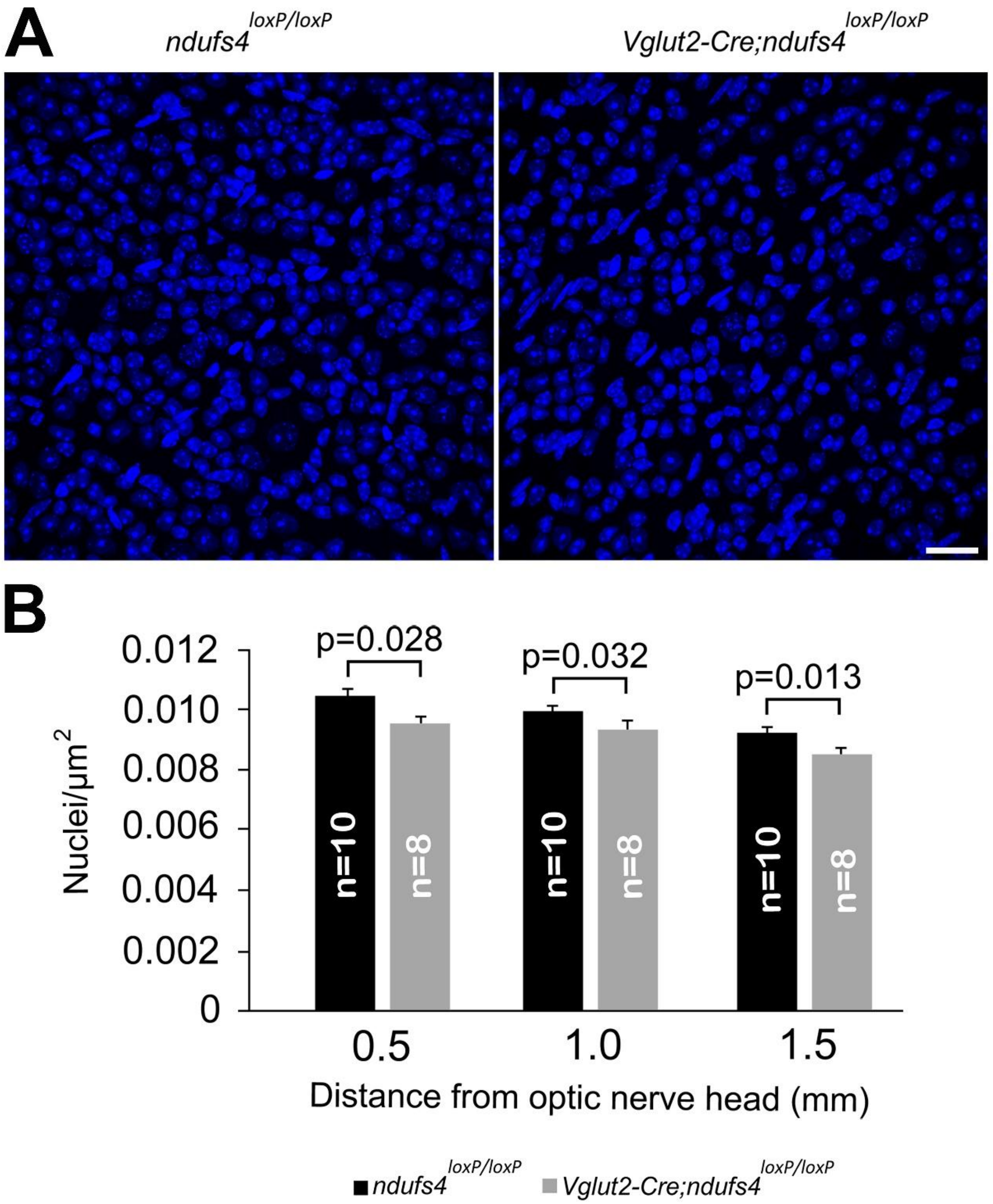


**Supplementary Figure S1.** Quantification of nuclear density within the ganglion cell layer of retinal flat mounts. (A) Retinal flat mounts from *ndufs4*$^{loxP/loxP}$ mice (left panel) and *Vglut2-Cre;ndufs4*$^{loxP/loxP}$ mice (right panel) at P60 were stained with DAPI to label nuclei. Representative images acquired at a location 1.5 mm from the optic nerve head are depicted. Scale bar, 20 µm. (B) Bar graphs comparing the density of nuclei for the indicated genotypes at distances of 0.5, 1.0, and 1.5 mm from the optic nerve head. Data depicted as mean ± SEM; the p-value for each comparison is shown above; n = number of retinas analyzed.

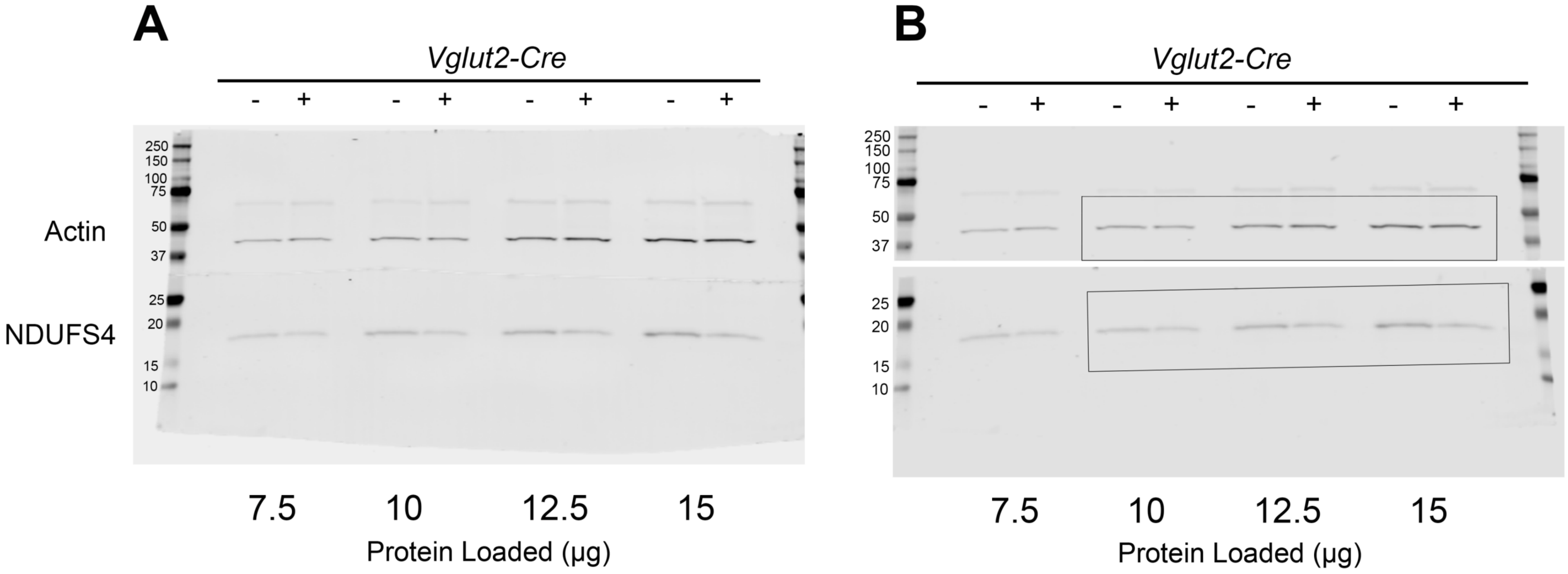


**Supplementary Figure S2.** Uncropped Western blot of optic nerve lysates from Fig. 3A. (A) Western blot comparing the expression of NDUFS4 (18 kDa) and actin (42 kDa) in optic nerve lysates from *ndufs4*$^{loxP/loxP}$ and *Vglut2-Cre; ndufs4*$^{loxP/loxP}$ mice at P30. Total protein content of lysate loaded into each lane is indicated below, and the presence or absence of the *Vglut2-Cre* transgene is indicated above each lane. After transfer, the membrane was cut between the 25-kDa and 37-kDa molecular weight markers and each half blotted with the indicated primary antibody. There is a non-specific ~70 kDa band which runs above the actin band. (B) Each half of the blot was scanned individually to optimize signal intensity for each protein. The portions of these blots that were included in Fig. 3A are marked by boxes. Because the lanes with 7.5 µg total protein produced faint NDUFS4 bands with signal outside the linear range, these were not included in Fig. 3A.
